# Identification of potential crucial genes and construction of microRNA-mRNA negative regulatory networks in osteosarcoma

**DOI:** 10.1186/s41065-018-0061-9

**Published:** 2018-05-09

**Authors:** Yue Pan, Lingyun Lu, Junquan Chen, Yong Zhong, Zhehao Dai

**Affiliations:** 1 Department of Spine Surgery, the Second Xiangya Hospital, Central South University, Changsha, 410011 China; 2 Department of Orthopaedics, the Fifth Hospital of Xiamen, Xiamen, 361101 China; 3 Department of Nephrology, Xiangya Hospital of Central South University, Changsha, 410008 China

**Keywords:** Osteosarcoma, Bioinformatics, Functional enrichment analysis, Regulatory network

## Abstract

**Background:**

This study aimed to identify potential crucial genes and construction of microRNA-mRNA negative regulatory networks in osteosarcoma by comprehensive bioinformatics analysis.

**Methods:**

Data of gene expression profiles (GSE28424) and miRNA expression profiles (GSE28423) were downloaded from GEO database. The differentially expressed genes (DEGs) and miRNAs (DEMIs) were obtained by R Bioconductor packages. Functional and enrichment analyses of selected genes were performed using DAVID database. Protein–protein interaction (PPI) network was constructed by STRING and visualized in Cytoscape. The relationships among the DEGs and module in PPI network were analyzed by plug-in NetworkAnalyzer and MCODE seperately. Through the TargetScan and comparing target genes with DEGs, the miRNA-mRNA regulation network was established.

**Results:**

Totally 346 *DEGs* and 90 DEMIs were found to be differentially expressed. These DEGs were enriched in biological processes and KEGG pathway of inflammatory immune response. 25 genes in the PPI network were selected as hub genes. Top 10 hub genes were TYROBP, HLA-DRA, VWF, PPBP, SERPING1, HLA-DPA1, SERPINA1, KIF20A, FERMT3, HLA-E. PPI network of DEGs followed a pattern of power law network and met the characteristics of small-world network. MCODE analysis identified 4 clusters and the most significant cluster consisted of 11 nodes and 55 edges. SEPP1, CKS2, TCAP, BPI were identified as the seed genes in their own clusters, respectively. The miRNA-mRNA regulation network which was composed of 89 pairs was established. MiR-210 had the highest connectivity with 12 target genes. Among the predicted target of MiR-96, HLA-DPA1 and TYROBP were the hub genes.

**Conclusion:**

Our study indicated possible differentially expressed genes and miRNA, and microRNA-mRNA negative regulatory networks in osteosarcoma by bioinformatics analysis, which may provide novel insights for unraveling pathogenesis of osteosarcoma.

## Background

Osteosarcoma is the most common primary malignant bone cancer in children and adolescents, which originates from mesenchymal stem cells and exhibits osteoblastic differentiation [1]. The incidence rate of osteosarcoma is approximately one to three cases per million each year worldwide [2]. With the development of surgery and chemotherapy, the survival rate in osteosarcoma patients without distal metastasis has been largely increased [3]. However, despite improvements in osteosarcoma therapy over the last three decades, the overall survival of patients has reached a plateau and about 30–40% of the patients experience progressive metastasis within 5 years after diagnosis and die [4]. Therefore, it is extremely necessary to explore novel biomarkers and therapeutic targets for osteosarcoma.

In recent years, the developments in molecular biology have provided some new insights into potential diagnostic and therapeutic biomarkers for osteosarcoma. For instance, it was found that SPARCL1 was downregulated in osteosarcoma by epigenetic methylation of promoter DNA and activating the expression of SPARCL1 could inhibit the osteosarcoma metastasis in vitro and in vivo [5]. NRP2 was reported to be up-regulated in osteosarcoma cell lines and tissues, and associated with poor survival of osteosarcoma patients [6]. On the other hand, numerous studies shows that microRNAs (miRNAs) may play essential roles in osteosarcoma tumorigenesis by negatively regulating expression level of target gene. MiR-497, for instance, can activate P21 expression by inhibiting the expression of MAPK/Erk signaling pathway, and promote the apoptosis of osteosarcoma cells [7]. However, the miRNA-mRNA negative regulation network in osteosarcoma had been not fully delineated by now.

In this study we used bioinformatics methods to integrate miRNA and mRNA expression data, which are available in the GEO database, to identify differentially expressed genes (DEGs) and miRNAs (DEMIs) between osteosarcoma and normal cell, and establish the miRNA-mRNA negative regulation network, aiming to provide valuable information for use in defining the mechanism of pathogenesis in osteosarcoma.

## Methods

### Identification of differentially expressed genes and miRNAs from public microarray data

To explore the DEGs and DEMIs in osteosarcoma compared to normal bone, the public gene expression (GSE28424) and miRNA expression (GSE28423) profiles were downloaded from the Gene Expression Omnibus (GEO, http://www.ncbi.nlm.nih.gov/geo). These profiles were deposited by Namløs HM et al. in 2011 and composed with the same cell lines: 19 OS cell lines (case group) and 4 normal bone samples (control group) [8]. Then, the dataset was analyzed by R Bioconductor packages and raw datasets were normalized based on the preprocess Core package and the DEGs and DEMIs were screened out via the limma package through the cut-off criteria of adjusted *P*-value< 0.01 and |Log_2_(FC)| > 2.

### Functional and pathway enrichment analysis

The Database for Annotation, Visualization and Integrated Discovery (DAVID, https://david.ncifcrf.gov/) was used to perform functional and pathway enrichment analysis. DAVID is a systematic and integrative functional annotation tools which allows investigators to unravel biological meaning behind large list of genes [9]. Gene ontology (GO) analysis including the cellular component (CC), molecular function (MF), and biological process (BP) [10] and Kyoto Encyclopedia of Genes and Genomes (KEGG) pathway enrichment analysis [11] were carried out for the DEGs. *P* < 0.05 was regarded as statistical significance.

### Protein–protein interaction (PPI) network construction and module analysis

In order to interpret the molecular mechanisms of key cellular activities in osteosarcoma, The online tool, Search Tool for the Retrieval of Interacting Genes database (STRING), was used to construct PPI network of the DEGs [12]. The interaction score of not < 0.7 (high confidence score) was considered significant and the PPI was visualized.

The relationships among the DEGs were analyzed by plug-in NetworkAnalyzer of Cytoscape software for the characteristics of small-world network through calculating the network properties such as distribution of network node degree, distribution of the shortest path, average aggregation coefficient and proximity to the center [13]. Subsequently, the hub genes were selected according to connection degree. Moreover, Molecular Complex Detection (MCODE) was applied to find clusters of genes in PPI network. “Degree cutoff = 2”, “node score cutoff = 0.2”, “k-core = 2” and “max. depth = 100” were set as the cut-off criterion.

### Prediction of miRNA targets

The target genes of DEMIs were predicted through the Targetscan (http://www.targetscan.org/), an online program that predicts targets of miRNAs by seeking the specific sequence complementary to the seed region of each miRNA. According to the predicted efficacy of targeting as calculated using cumulative weighted context++ scores of the sites, predicted targets are ranked. In this study, the genes with the cumulative weighted context++ scores ≤ − 0.4 were selected as target genes of each miRNA. Furthermore, we only selected the reverse pairs that contained the DEMIS and overlapping genes of DEGs. Finally, miRNA–mRNA negative regulatory network depicting interactions between miRNAs and their potential targets in osteosarcoma was visualized using Cytoscape.

## Results

### Identification of DEGs and DEMIs

Compared with normal bone samples, a total of 346 *DEGs* were identified in the osteosarcoma cells, which contained 43 up-regulated and 303 down-regulated genes. The top ten up-regulated and down-regulated genes are listed in Table 1. In total, 90 DEMIs were found to be differentially expressed. 58 DEMIs were downregulated and 32 DEMIs were upregulated. The top ten significantly differentially expressed miRNAs are showed in Table 2.

**Table 1:** The most significant up-regulated and down-regulated genes

Gene symbol	Log2(FC)	adj.P.Val
up-regulated		
CBS	3.44	4.06E-05
TMSL8	3.35	0.004377385
PSAT1	3.20	0.002425845
PHGDH	3.07	2.24E-06
ASNS	2.91	0.0002163
TUBB3	2.73	0.006893245
MGC39900	2.63	0.003410321
UBE2C	2.62	0.000193362
PBK	2.51	9.68E-05
LARP6	2.48	0.00171183
down-regulated		
HBB	−7.79	5.88E-34
HBA1	−7.33	2.86E-23
MMP9	−6.34	1.09E-10
CD74	−5.84	1.03E-16
S100A8	−5.83	1.21E-08
VWF	− 5.64	1.07E-11
HLA-DRA	−5.54	1.27E-10
LYZ	−5.43	2.53E-12
C1QA	−5.28	6.33E-25
TYROBP	−5.27	1.17E-21

**Table 2:** The most significantly differentially expressed miRNAs

miRNA	Log2(FC)	adj.P.Val
hsa-miR-451	−15.39	2.80E-07
hsa-miR-144	−9.27	9.93E-09
hsa-miR-142-3p	−8.33	0.000179008
hsa-miR-223	−7.52	3.60E-05
hsa-miR-126	−6.35	4.93E-05
hsa-miR-142-5p	−6.12	2.81E-07
hsa-miR-9	6.04	0.00168538
hsa-miR-18a	6.03	0.000307596
hsa-miR-150	−5.94	0.000189034
hsa-miR-486-5p	−5.20	2.25E-05

### GO functional annotation and pathway enrichment

**Fig. 1:**
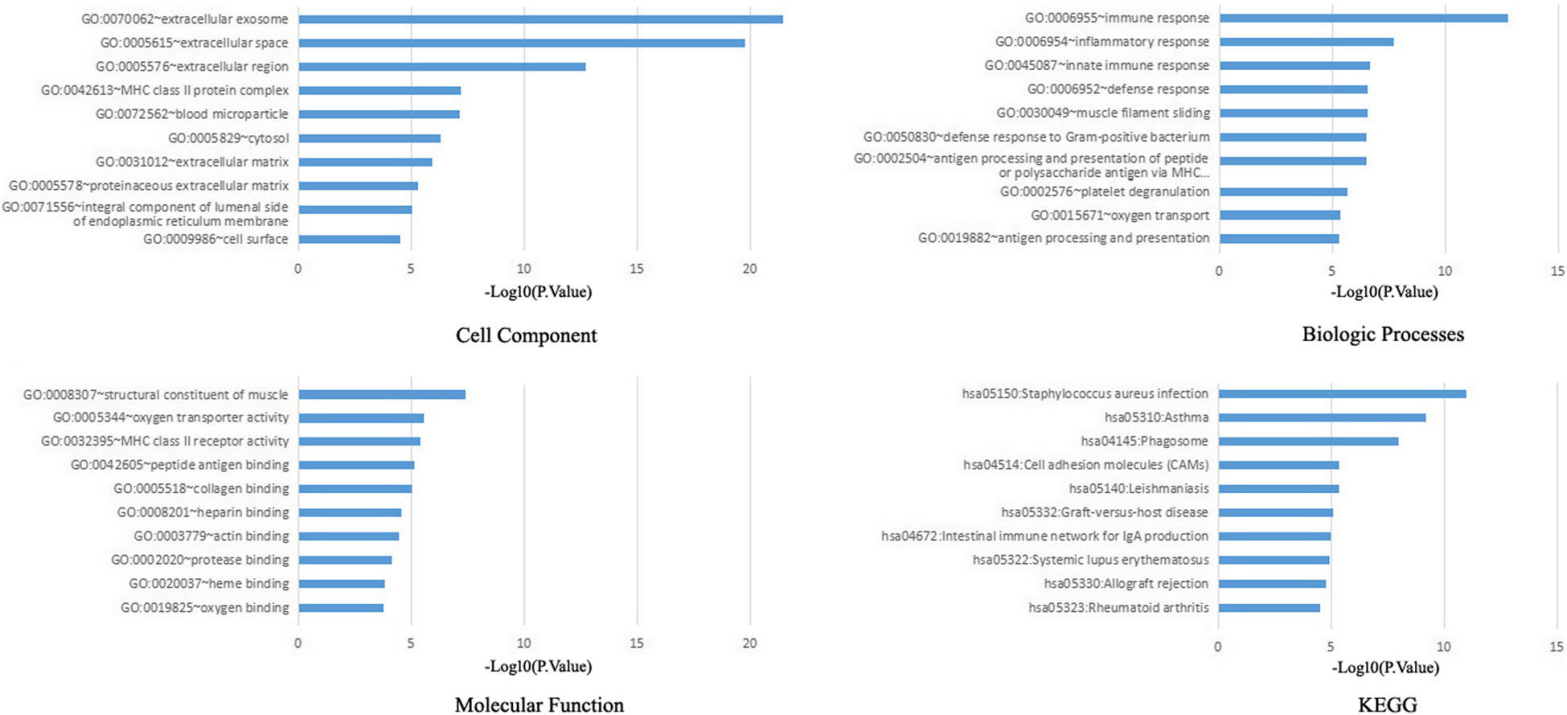
Gene ontology and pathway enrichment analysis of the differentially expressed genes in osteosarcoma

The top 10 significant terms of GO functional annotation and pathway enrichment analysis in DAVID were illustrated as Fig. 1.

In the CC ontology, we found that the majority of enriched categories were relevant to extracellular components, such as extracellular exosome(120 genes), extracellular space (77 genes), extracellular region (72 genes), and extracellular matrix (20 genes). The second majority of enriched categories were associated with cytosol (95 genes). In addition, the other enriched CC GO terms contained cell surface (25 genes), MHC class II protein complex (8 genes) and proteinaceous extracellular complex (18 genes). In the BP ontology, the regulation of inflammatory immune response items constitute the majority of enriched GO categories, including immune response (35 genes), innate immune response (26 genes), inflammatory response (26 genes), defense response to Gram-positive bacterium (12 genes), defense response(11 genes) and antigen processing and presentation (9 genes) and antigen processing and presentation of peptide or polysaccharide antigen via MHC class II (7 genes). The other enriched BP GO terms contained platelet degranulation (12 genes), muscle filament sliding (9 genes)and oxygen transport (6 genes). In the MF ontology, the binding-related items constitute the majority of enriched GO categories, including actin binding (17 genes), heparin binding (13 genes), heme binding (11 genes), protease binding (10 genes), collagen binding (9 genes), peptide antigen binding (7 genes) and oxygen binding (7 genes). Besides, the other enriched categories comprised items related to structural constituent of muscle (10 genes), oxygen transporter activity (6 genes) and MHC class II receptor activity (6 genes).

Furthermore, the KEGG pathways of DEGs mainly involved in inflammatory immune response, which included Phagosome (19 genes), *Staphylococcus aureus* infection (15 genes), Cell adhesion molecules (CAMs) (15 genes), Systemic lupus erythematosus (14 genes), Asthma (11 genes) and et al.

### PPI network construction, module analysis and hub gene selection

**Fig. 2:**
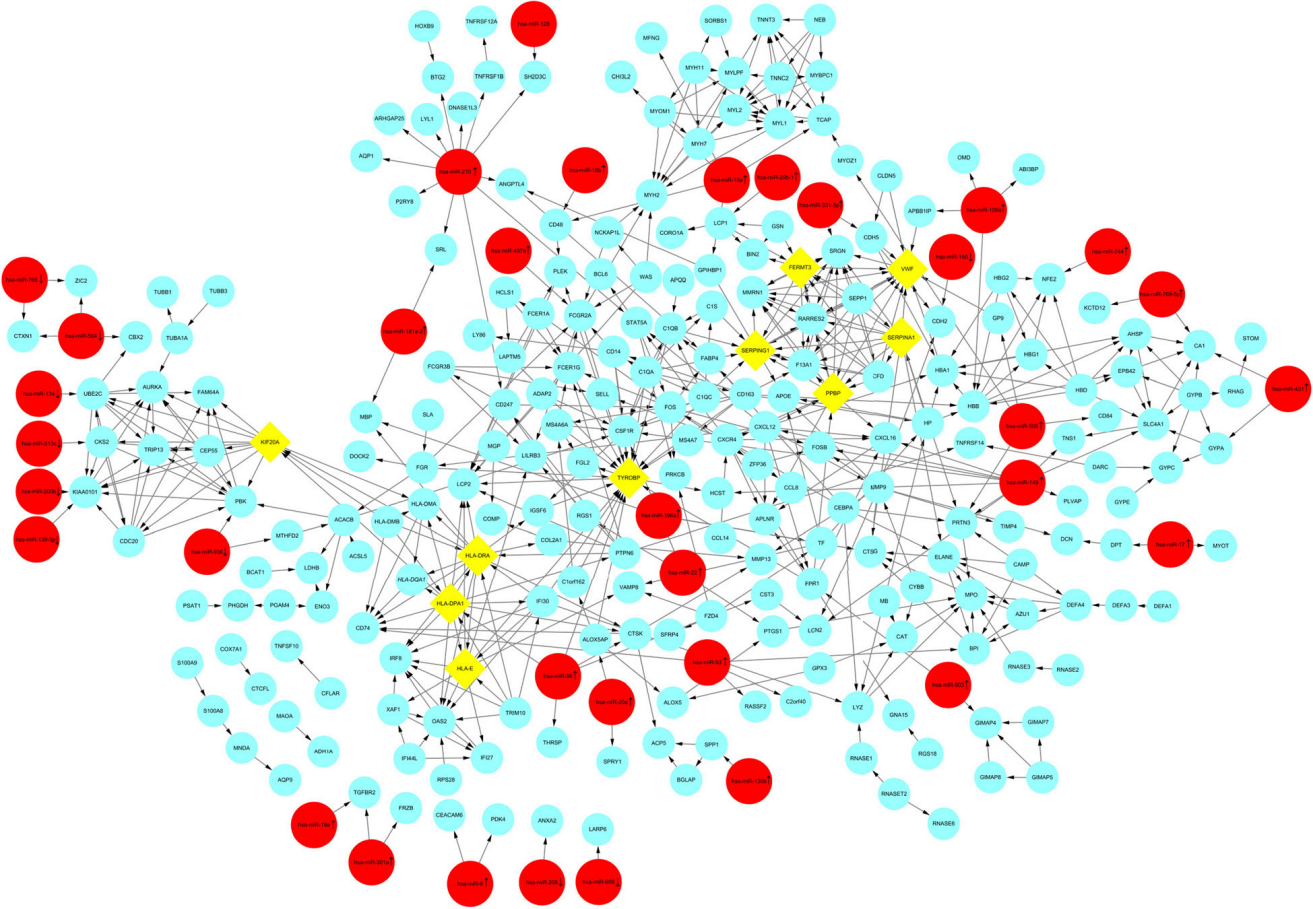
The protein-protein interaction network for the the differentially expressed genes and miRNA-target gene regulatory network in osteosarcoma. Circular nodes in light blue represent the differentially expressed genes; diamond nodes in yellow represent the hub genes; Circular nodes in red represent the screened out differentially expressed miRNAs and arrows in nodes represent the change trend of miRNAs

**Fig. 3:**
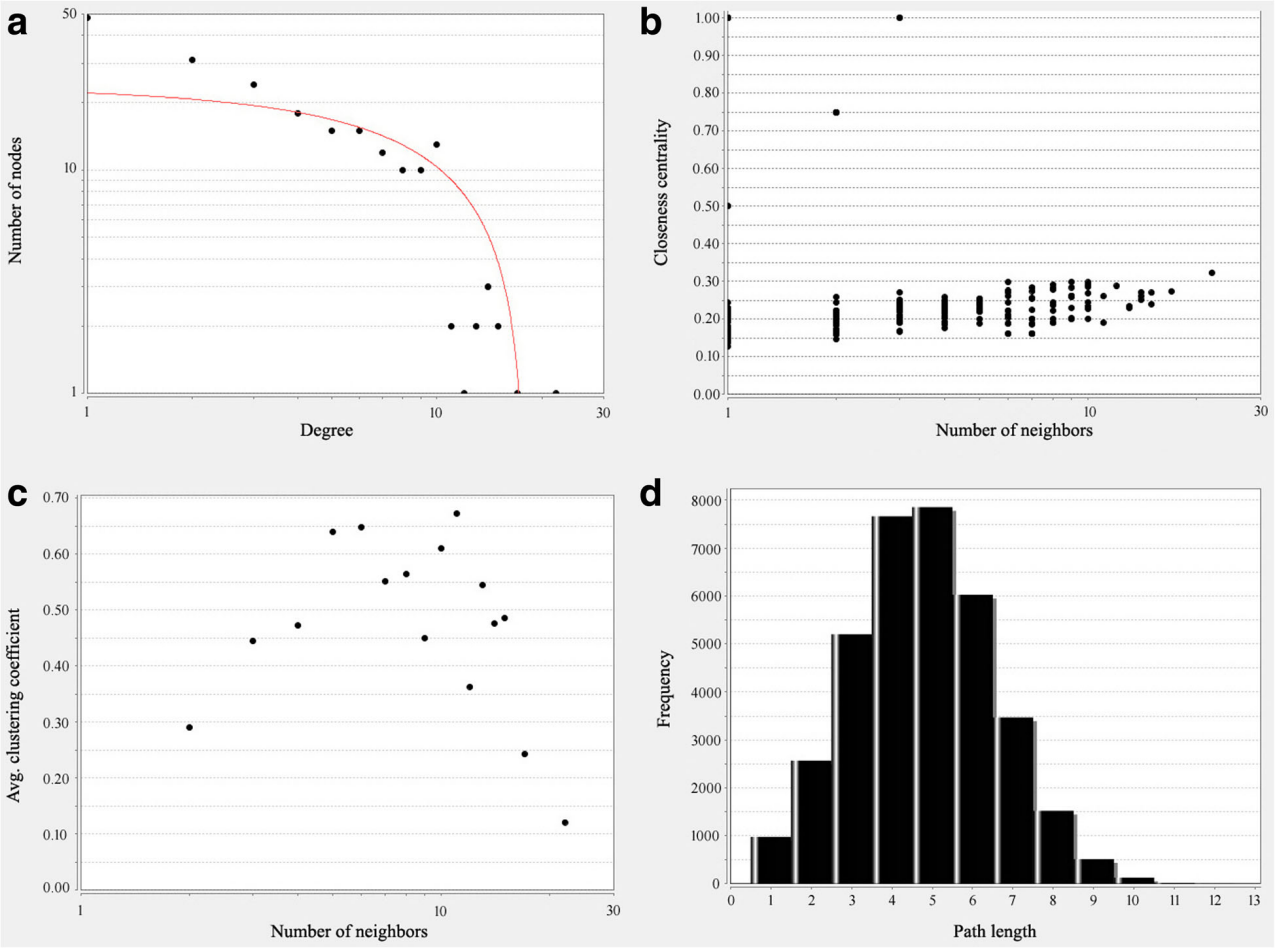
PPI network analysis of DEGs for characteristics of small-world network. (**a**) Distribution of degrees (**b**) the proximity to the centre (**c**) average aggregation coefficient (**d**) distribution of the shortest path

PPI networks were constructed on the basis of STRING database and displayed in Fig. 2. We also analyzed the network properties, as shown in Fig. 3a–d. We could see the distribution of network node degrees followed a pattern of power law network in Fig. 3a, proximity to center in Fig. 3b, the average clustering coefficient in Fig. 3c and fromthe shortest path in Fig. 3d and we could also see that they meet the characteristics of small world network. When “Degree≥10” was set as the cut-off criterion, 25 genes in the PPI network were selected as hub genes in osteosarcoma. Top 10 hub genes were TYROBP, HLA-DRA, VWF, PPBP, SERPING1, HLA-DPA1, SERPINA1, KIF20A, FERMT3, HLA-E, and showed in Fig. 2. These hub genes might play crucial roles in osteosarcoma.

**Fig. 4:**
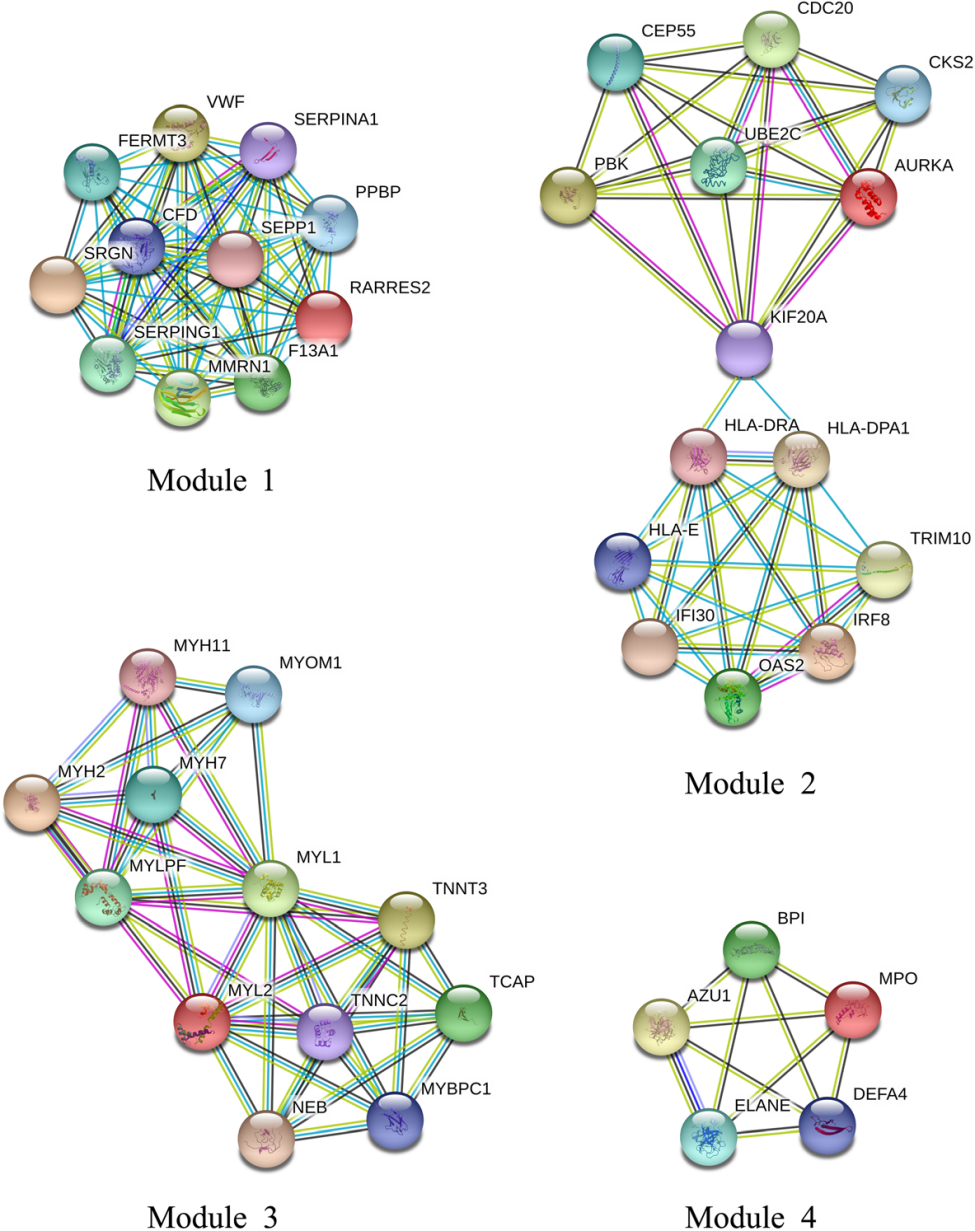
Four significant modules selected from protein-protein interaction network

Subsequently, when “score ≥ 5” was defined as the cut-off criterion in MCODE, 4 clusters were identified from PPI network, and the most significant cluster consisted of 11 nodes and 55 edges. Furthermore, MCODE analysis showed that each cluster contained one seed gene. SEPP1 (one of the hub genes), CKS2 (the predicted target of MiR-513c), TCAP (one of the predicted target of MiR-18a), BPI (one of the predicted target of MiR-93) were identified as the seed genes in their own clusters, respectively [Fig. 4].

### miRNA-mRNA negative regulation network

Through the TargetScan, the target genes of 90 DEMIs were predicted. By comparing target genes with DEGs, we screened out 35 DEMIs (25 upregulated and 10 downregulated miRNA) and 78 DEGs (67downregulated and 11 upregulated mRNA), presented an expression trend opposite to each other in osteosarcoma. And then, the miRNA-mRNA regulation network which was composed of 89 pairs was established and shown in Fig. 2. In our study, miR-9, the most significantly upregulated among these screened out DEMIS, was predicted to 2 downregulated target, including CEACAM6 and PDK4. MiR-210 was with the highest connectivity with target genes, targeting 12 differentially expressed genes (ANGPTL4, AQP1, ARHGAP25, BTG2, CD247, DNASE1L3, LYL1, P2RY8, SH2D3C, SRL, STAT5A, TNFRSF1B), that might indicate its important role in osteosarcoma. Among the predicted target of MiR-96, HLA-DPA1 and TYROBP were the hub genes. Moreover, several genes were predicted to the common targets of different miRNA. For instance, CSF1R, one of the hub genes, was predicted as the common target of miR-149 and miR-22.

## Discussion

Unraveling the mechanisms of the initiation and development of osteosarcoma would benefit the diagnosis, treatment and prognosis evaluation. In present study, two types of microarray datasets: gene expression profile and miRNA expression profile were downloaded from the GEO and analyzed for their relationship in osteosarcoma. Compared with normal bone samples, a total of 346 *DEGs* were identified in the osteosarcoma cells, containing 43 up-regulated and 303 down-regulated genes. CBS (cystathionine-β-synthase cancer), the most regulated gene in this study, was found to be upregulated in many types of tumors, such as multiple myeloma [14], colorectal cancers [15], bladder cancer [16] and et al., and downregulation of CBS help inhibit carcinogenesis. However, to our knowledge, there was no report about CBS in osteosarcoma, so it might be a novel target for the diagnosis or treatment of osteosarcoma.

As was suggested by DAVID analysis, the DEGs in osteosarcoma were enriched in biological processes and KEGG pathway of inflammatory immune response. Molecular function of GO analysis showed enrichment in binding-related items. It is reasonable because immune system has significant roles in tumorigenesis. At the cancer initiation stage, the immune system can recognize tumors expressing foreign antigens and eradicate tumors via innate and adaptive immune activation [17]. If tumor cells are not eliminated via recognition by the immune system, tumor cells can enter an equilibrium phase and evolve a number of phenotypic changes and dampen immunogenicity to escape the immune surveillance [18, 19]. In other words the immune microenvironment helps cancer cells to select the dominant cells so that the tumor can progressively grow unhindered. Therefore, osteosarcoma tumors are infiltrated with immune cells that may have the ability to fight tumor cells [20] but are tolerized due to immune escape of the tumor cells [21, 22]. Cellular component of GO analysis showed the majority of enriched categories were relevant to extracellular components, such as extracellular exosome, extracellular space, extracellular region, and extracellular matrix. Tumor microenvironment has complementary effects on the development and metastasis of osteosarcoma through extracellular secretion, alteration of phenotype type of tumor cells, immune escape and providing proper acid-base environment for tumor cells [23].

PPI network of DEGs illustrated the overview of their functional connections, which followed a pattern of power law network from the shortest path, average aggregation coefficient, node degree and proximity to the center and met the characteristics of small-world network, and of which 25 hub genes were selected. Most of them were enriched in inflammatory immune response and platelet degranulation. As we known, the activated degranulated platelet accumulated on the surface of tumor cell to protect them from the immune system recognization and eradication. After module analysis of the PPI network, 4 seed genes were selected, such as SEPP1, CKS2, TCAP and BPI genes.

SEPP1, a major selenium transport protein, has endogenous antioxidant function through catalyzing the oxidation of glutathione by a hydrogen peroxide or phosphatidylcholine hydroperoxide [24]. It was found that SEPP1 levels and activity are significantly decreased in colon tumors, human prostate tumors, C3(1)/Tag transgenic ouse tumors, and prostate cancer cell lines [25, 26]. Furthermore, several single nucleotide polymorphisms (SNPs) have been identified in SEPP1 that may contribute to decreased expression in colorectal adenomas and have been associated with cancer risk [27, 28]. CKS2 (Cyclin-dependent kinases regulatory subunit 2), a cyclin-dependent kinase interacting protein, is essential for cell cycle regulation. Elevated expression of CKS2 has been demonstrated in multiple types of human malignancies. In prostate cancer, aberrant expression of CKS2 contributes to tumorigenesis by enhancing cell proliferation and inhibiting programmed cell death [29]. In a papillary thyroid carcinoma, miR-26a [30] and miR-7 [31] modulates tumor growth and tumorigenesis by targeting CKS2. In colorectal cancer cells, attenuation of CKS2 results in decreased cell viability, increased cell apoptosis and cell cycle arrest [32]. In almost the same way, aberrant expression of TCAP and BPI was in relation with multiple types of tumor. However, no study has associated these seed genes with osteosarcoma, which might indicate them as the new targets.

It has been shown that miRNAs induce RNA silencing by targeting 3’-UTR of mRNAs, and that miRNA functions as oncogenes or oncosuppressor depending on the function of suppressed targets. In present study, the miRNA-mRNA regulation network which was composed of 89 pairs was established through the TargetScan and comparing target genes with DEGs, in which MiR-210 directly regulated 12 differentially expressed genes and was significantly upregulated in osteosarcoma. Mounting evidence identified MiR-210 as an oncogenic role in generating osteosarcoma; increased expression of MiR-210 is associated with decreased overall survival and progression-free survival, and more frequently occurred in osteosarcoma tissues with large tumor size, poor response to preoperative chemotherapy, and positive metastasis [33, 34]. Nonetheless, the defined roles of miR-210 in osteosarcoma malignant progression, especially the target gene of miR-210 in osteosarcoma dedifferentiation, was insufficiently researched. Only NFIC, a validated target of miR-210 from miRBase and a DEG from a gene expression profile GSE38135, had been shown to play important role in TGF-b1-induced osteosarcoma dedifferentiation and can be significantly reduced by miR-210 treatment in human osteosarcoma cell line MNNG/HOS [35]. So far, the role of other miR-210-gene pairs in osteosarcoma was not reported. HLA-DPA1 and TYROBP, the hub genes in PPI network were regulated by MiR-96, but their function were not researched in osteosarcoma too. These might imply that miRNA and their target genes may represent potential novel therapeutic targets or biomarkers for osteosarcoma.

The limitation in our work is that the pathogenesis of key miRNA and gene in osteosarcoma need to be elucidated through experiments in vivo and in vitro.

## Conclusion

In summary, our study was intended to identify key genes in osteosarcoma and construct regulatory networks between miRNA and mRNA through bioinformatics analysis. 25 hub genes and 4 seed genes were identified according to PPI network.

Functional and pathway enrichment analysis indicated immune system played significant roles in osteosarcoma tumorigenesis. Additionally, deregulated miRNAs such as MiR-210 and MiR-96, might exert their biological functions through their targeting mRNAs. Moreover, our results could provide novel sights in the mechanisms of the initiation and development of osteosarcoma, that would become the new diagnostic biomarkers and treatment targets for osteosarcoma.

## Abbreviations

BP: Biological processes
CC: Cell component
DEG: Differentially expressed gene
DEMI: Differentially expressed miRNAs
GO: Gene ontology
KEGG: Kyoto Encyclopedia of Genes and Genomes
MF: Molecular function
PPI: Protein–protein interaction
STRING: Search Tool for the Retrieval of Interacting Genes
TCGA: The Cancer Genome Atlas
